# Data on the implementation of VaxTrac electronic immunization registry in Sierra Leone

**DOI:** 10.1016/j.dib.2020.106167

**Published:** 2020-08-12

**Authors:** Apophia Namageyo-Funa, Mohamed F Jalloh, Brigette Gleason, Aaron S Wallace, Michael Friedman, Tom Sesay, Dennis Ocansey, Mohamed S Jalloh, Leora R Feldstein, Laura Conklin, Sara Hersey, Tushar Singh, Reinhard Kaiser

**Affiliations:** a Strategic Information and Workforce Development Branch, Global Immunization Division, U.S. Centers for Disease Control and Prevention, Atlanta, United States; b Immunization Systems Branch, Global Immunization Division, U.S. Centers for Disease Control and Prevention, Atlanta, United States; c Sierra Leone Country Office, Division of Global Health Protection, U.S. Centers for Disease Control and Prevention, Freetown, Sierra Leone; d Sierra Leone Ministry of Health and Sanitation, Freetown, Sierra Leone; e eHealth Africa, Freetown, Sierra Leone

**Keywords:** Assessment, Data source, Health facility, Sierra Leone, Vaccine, VaxTrac

## Abstract

Following the piloting of VaxTrac, an electronic immunization registry (EIR), we conducted a rapid assessment in November-December 2017 to evaluate the use of the EIR in 10 health facilities in Western Area Urban district in Sierra Leone [1]. In this data-in-brief report, we provide additional descriptive data from the assessment of the VaxTrac EIR in Sierra Leone. The assessment comprised aggregate data on vaccine doses administered that were abstracted from VaxTrac and three paper-based sources (daily tally sheets, register of children under the age of 2 years, and a summary form of doses administered). Data were abstracted for the following six vaccine doses in the immunization schedule in Sierra Leone: 1) Bacillus Calmette–Guérin vaccine, 2) first dose of pentavalent vaccine, 3) second dose of pentavalent vaccine, 4) third dose of pentavalent vaccine, 5) first dose of measles-containing vaccine, and 6) second dose of measles-containing vaccine. We descriptively analysed the abstracted data to examine the congruity between VaxTrac records and the three paper-based sources. Bar graphs were generated to visually depict the variations in number of administered vaccine doses by data source for each health facility. We provide the aggregated data for each vaccine dose abstracted by data source from each health facility as supplemental material (Excel file). The supplementary data reveal patterns in the congruity of vaccine doses captured that have implications for policy and programmatic decisions regarding the use of VaxTrac and other similar EIRs in low resource urban settings.

**Specifications Table**

**Table:** 

**Subject**	Health Informatics.
**Specific subject area**	Immunization and Public health.
**Type of data**	Figures Microsoft Excel file
**How data were acquired**	Cross-sectional survey administered and data abstraction in 10 health facilities.
**Data format**	Raw data abstracted from 4 data sources within a health facility.
**Parameters for data collection**	Health facilities that provide and capture data on immunization services.
**Description of data collection**	Data were abstracted from paper-based tools and VaxTrac an electronic based tool at 10 health facilities.
**Data source location**	Wester Urban district in Sierra Leone – National Expanded Program on Immunization and Ministry of Health and Sanitation
**Data accessibility**	Data is available with the article.
**Related research article**	Jalloh MF, Namageyo-Funa A, Gleason B, et al. Assessment of VaxTrac electronic immunization registry in an urban district in Sierra Leone: Implications for data quality, defaulter tracking, and policy [published online ahead of print, 2020 Aug 1]. Vaccine. 2020; S0264-410X(20)30949-X. doi:10.1016/j.vaccine.2020.07.031

## 1 Value of the Data

• The data can be used to examine the completeness of vaccine doses captured in immunization registers in urban health facilities in the context of introducing an electronic registry alongside existing paper-based registers.
• The data can be used to understand patterns in the reported utilization of immunization services by source (paper-based versus electronic).
• The data provides insights on data quality considerations regarding the use of an electronic immunization registry to capture vaccine doses administered in urban health facilities in a low resource setting.
• The data can help to guide Expanded Programs on Immunization on the feasibility of using VaxTrac to identify and reach children who have missed one or more scheduled vaccine dose(s).

## 2 Data Description

**Fig. 1:**
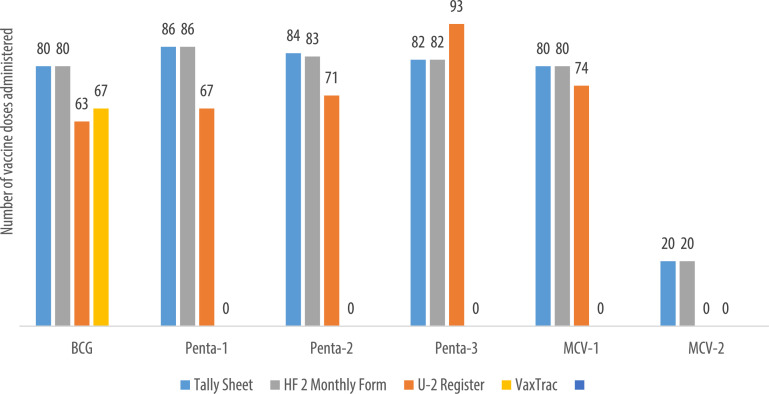
Distribution of vaccine doses administered in Military 34 Hospital by data sources.

**Fig. 2:**
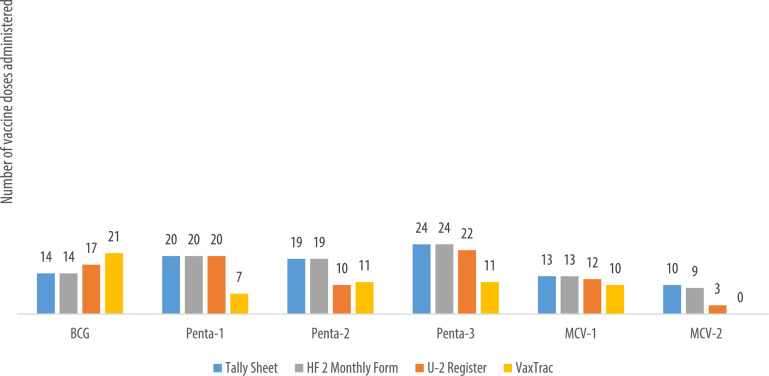
Distribution of vaccine doses administered in Allen Town Community Health Center by data sources.

**Fig. 3:**
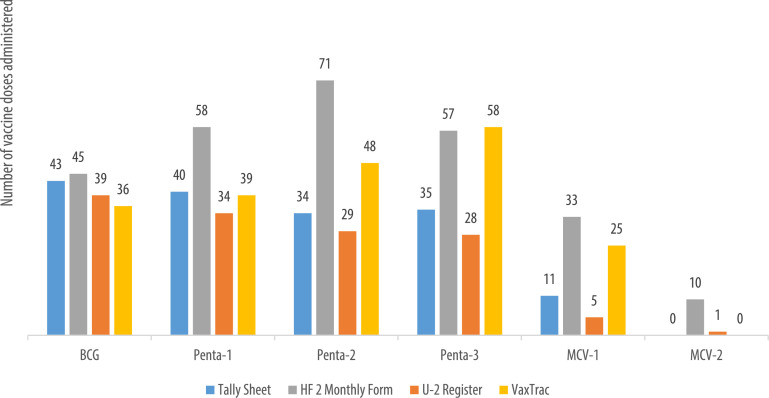
Distribution of vaccine doses administered in Calaba Community Health Center by data sources.

**Fig. 4:**
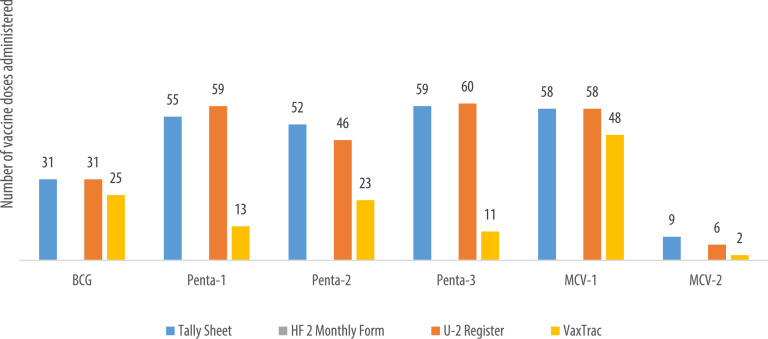
Distribution of vaccine doses administered in Murray Town Community Health Center by data sources.

**Fig. 5:**
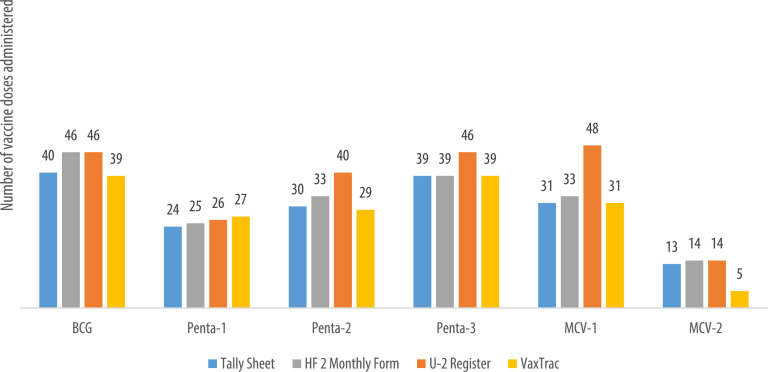
Distribution of vaccine doses administered in King Harman Hospital by data sources.

**Fig. 6:**
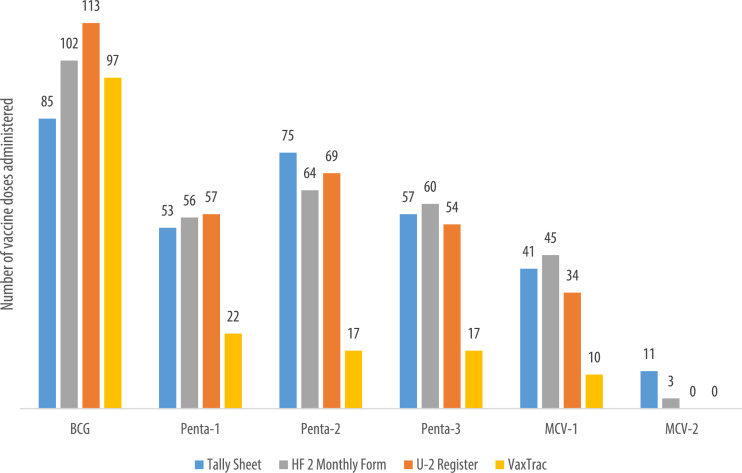
Distribution of vaccine doses administered in Rokupa Hospital by data sources.

**Fig. 7:**
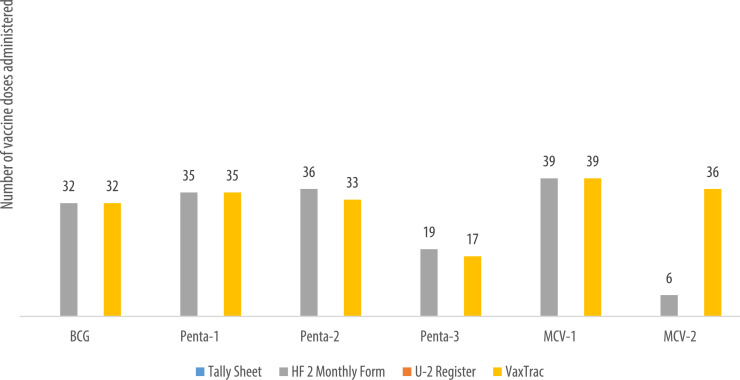
Distribution of vaccine doses administered in Mabella Community Health Center by data sources.

**Fig. 8:**
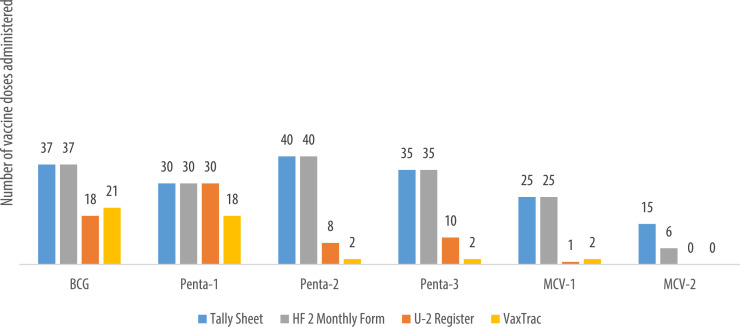
Distribution of vaccine doses administered in Kuntorloh Community Health Center by data sources available.

**Fig. 9:**
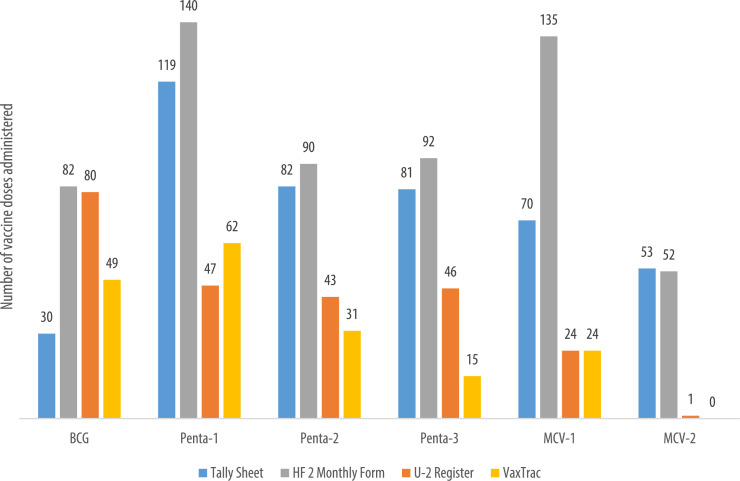
Distribution of vaccine doses administered in Kissy Community Health Center by data sources available.

We describe data on the number of vaccine doses administered by data source in 9 of the 10 health facilities. Data from 1 of the 10 health facilities is reported elsewhere [1]. The raw data were abstracted from three paper-based sources: tally sheets, under-two registers, Health Facility Form 2 [HF2], and VaxTrac. The reported number of vaccine doses administered in the health facilities were largely identical for the HF2 and tally sheets data sources in Military 34 Hospital (Fig. 1), Allen Town Community Health Center (Fig. 2), King Harman Hospital (Fig. 5) and Kuntorloh Community Health Center (Fig. 8). At the time of data collection, no data from the HF2 was available at Murray Town Community Health Center (Fig. 4). In this facility, the reported number of vaccine doses were largely similar in the tally sheets and the under-two register with fewer doses reported in VaxTrac. No data was available from the tally sheets or under-two register for Mabella Community Health Center (Fig. 7). In this facility, the reported number of vaccine doses administered in the HF2 was aligned with that reported in VaxTrac. There was no clear consistency in the number of vaccine doses administered by data source for Calaba Community Health Center (Fig. 3), Rokupa Hospital (Fig. 6), and Kissy Community Health Center (Fig. 9). The raw data for the bar charts is provided as a supplemental file – an Excel file.

## 3 Experimental Design, Materials and Methods

The rapid assessment was conducted in Western Area Urban district in Sierra Leone in 2017. Ten health facilities implementing VaxTrac were purposively selected to ensure variability in facility type, time since staff were trained, and geographic dispersion. Four hospitals and 6 community health centers that had implemented the VaxTrac system for a year were included in the assessment.

## 4 Data Collection

We abstracted facility-level aggregated doses administered for Bacillus Calmette–Guérin vaccine (BCG), first dose of pentavalent vaccine (Penta1), second dose of pentavalent vaccine (Penta2), third dose of pentavalent vaccine (Penta3), first dose of measles-containing vaccine (MCV1), and second dose of measles-containing vaccine (MCV2). To ensure that data had already been captured and/or aggregated at the facility-level, we allowed for a 2-month lag period. We therefore abstracted data for the month of September 2017 for the first batch of facilities visited in November 2017, and abstracted data for October 2017 for the second batch of facilities visited in December 2017. Monthly doses administered for the aforementioned vaccines were abstracted from three paper-based sources (tally sheets, under-two registers, and HF2) and VaxTrac.

## 5 Data Analysis

Quantitative data abstracted from facility-level sources were descriptively analyzed to examine congruity between VaxTrac records and paper-based data sources (tally sheets, under-two registers, and HF2) using Microsoft Excel version 2016’s PivotTable. Two facilities had one or more data sources missing: 1 facility was missing its HF2 summary form and a second facility was missing its tally sheet and under-two register. Number of monthly doses administered for BCG, Penta1, Penta2, Penta3, MCV1, and MCV2 were aggregated for each of the four data sources. To examine data congruity, we compared the cumulative doses administered between VaxTrac and the paper-based sources. Bar graphs were generated to visually depict the variations by vaccine dose and data source.

## 6 Ethics Statement

The assessment was approved as a public health program activity and received non-research determination from the Centers for Disease Control and Prevention Institutional Review Board (tracking # 2018-324). All respondents provided verbal consent before participating in the assessment. Permission to abstract the health facility data was obtained from the national manager of the Expanded Program on Immunization and respective health facility in-charges.

## Declaration of Competing Interest

The authors declare that they have no known competing financial interests or personal relationships which have, or could be perceived to have, influenced the work reported in this article.

## Appendix Supplementary materials

Image, application 1

## References

[1] Jalloh MF, Namageyo-Funa A, Gleason B (2020). Assessment of VaxTrac electronic immunization registry in an urban district in Sierra Leone: implications for data quality, defaulter tracking, and policy [published online ahead of print, 2020 Aug 1]. Vaccine.

